# δ-Opioid Receptor and Somatostatin Receptor-4 Heterodimerization: Possible Implications in Modulation of Pain Associated Signaling

**DOI:** 10.1371/journal.pone.0085193

**Published:** 2014-01-08

**Authors:** Rishi K. Somvanshi, Ujendra Kumar

**Affiliations:** Faculty of Pharmaceutical Sciences, The University of British Columbia, Vancouver, Canada; Temple University, United States of America

## Abstract

Pain relief is the principal action of opioids. Somatostatin (SST), a growth hormone inhibitory peptide is also known to alleviate pain even in cases when opioids fail. Recent studies have shown that mice are prone to sustained pain and devoid of analgesic effect in the absence of somatostatin receptor 4 (SSTR4). In the present study, using brain slices, cultured neurons and HEK-293 cells, we showed that SSTR4 and δ-Opioid receptor (δOR) exist in a heteromeric complex and function in synergistic manner. SSTR4 and δOR co-expressed in cortical/striatal brain regions and spinal cord. Using cultured neuronal cells, we describe the heterogeneous complex formation of SSTR4 and δOR at neuronal cell body and processes. Cotransfected cells display inhibition of cAMP/PKA and co-activation of SSTR4 and δOR oppose receptor trafficking induced by individual receptor activation. Furthermore, downstream signaling pathways either associated with withdrawal or pain relief are modulated synergistically with a predominant role of SSTR4. Inhibition of cAMP/PKA and activation of ERK1/2 are the possible cellular adaptations to prevent withdrawal induced by chronic morphine use. Our results reveal direct intra-membrane interaction between SSTR4 and δOR and provide insights for the molecular mechanism for the anti-nociceptive property of SST in combination with opioids as a potential therapeutic approach to avoid undesirable withdrawal symptoms.

## Introduction

The functional consequences of GPCRs heterodimerization in a native system expressing these receptors endogenously, specifically in the central nervous system (CNS) are poorly understood. Opioid receptors (ORs), namely mu (μ), delta (δ) and kappa (κ), are the prominent members of the GPCRs super family [1], [2]. The most indispensable function of ORs in CNS is to modulate pain. The activation of ORs in the presence of peptide produced endogenously or administered exogenously displayed distinct behavioural outcomes [3], [4]. µOR is believed to mediate antinociception associated with morphine, while δOR appears to participate in acute and tonic pain models [2], [5]–[7]. µOR is more efficient as an analgesic drug target due to its high expression at cell surface, however, reinstating δOR expression at neuronal membrane enhances receptor mediated analgesic effects [8]. These studies collectively suggest that ORs membrane expression is a prerequisite for receptors analgesic properties [8]. Interestingly, studies have also shown that knocking down δOR resulted in increased chronic pain and abolition of opioid mediated analgesic effects [9]. Furthermore, ORs functionally interact with other receptor of the family and display distinct pharmacological and signaling properties [10].

Like opioids, somatostatin (SST), is well expressed in the CNS and functions as a neurotransmitter and neuromodulator. In addition to exerting an inhibitory role on cell proliferation and hormone secretion, SST also plays a critical role in pain and inflammation [11], [12]. Intrathecal or epidural application of SST analogue octreotide (OCT) induced analgesic effects in post-operative and neoplastic pain [13]–[15]. SST analogues have also been used successfully for pain relief in conditions like headache or in patients with terminal cancer, where opioids failed [11], [12], [16]–[20]. Further, results from animal studies favour the role of SST in morphine sparing and analgesia [13]–[15]. The biological function of SST is mediated by binding to five different receptor subtypes namely somatostatin receptor 1–5 (SSTR1–5) [21]. Previous studies have shown that amongst all SSTRs, SSTR4 is the only subtype that mediates analgesic effects of SST. Neurogenic and non-neurogenic inflammatory processes were significantly reduced upon administration of SSTR4 specific agonist in animal models [22]. Recently, SSTR specific knockout (*ko*) models have provided new insights for the role of SSTRs in certain pathophysiological conditions such as inflammation and analgesia [22], [23]. Helyes et al., have described that SSTR4 *ko* mice are more susceptible to inflammation and exhibit sustained pain than *wt* mice [23].

OR and SSTR subtypes share >40% structural similarities, are coupled to pertussis toxin (PTX)-sensitive Gα_i/o_ subunits and inhibit the second messenger cAMP [2], [21], [24]–[27]. Previous studies have also described that OR and SSTR subtypes functionally interact with each other in heterologous systems and modulate receptor pharmacology and trafficking [28]. Furthermore, SST analogues exhibit the displacement of opiate binding in rat brain membrane suggesting the ability of SST to bind and activate ORs [29]–[35]. These are compelling pieces of evidence supporting the notion that SSTR and OR subtypes might functionally interact in a native system. Clinically, opioids are still the first line of therapy and the most dependable analgesic drugs in pain treatment; however, they are associated with several side effects including dependence and withdrawal. Whether, the use of SST analogs in combination with opioids minimize such risk factors is not known. To test this hypothesis, the present study was undertaken to elucidate the molecular details and functional consequences of a possible crosstalk between SSTR4 and δOR in rat brain slices, cultured neuronal cells and SSTR4/δOR cotransfected HEK-293 cells using morphological, biophysical and biochemical techniques.

As stated above, µOR is the prominent receptor subtype linked with pain relief and its pronounced analgesic effects, whereas, the role of δOR is also well appreciated in anti-nociception. In acute and chronic animal pain models, δOR agonists induced anti-nociceptive responses [36]–[38]. Mice lacking δOR are highly susceptible to pain and restoring δOR membrane expression is required to exert pain relieving and analgesic effect. δOR knockout mice displayed enhanced mechanical and thermal allodynia, and thermal hyperalgesia [9], [39]. Therefore, in the present study we preferred δOR over µOR to ascertain whether SSTR4 enhances δOR function, expression and signaling pathways which has not been explored yet. Here, we provide direct evidence that SSTR4 and δOR exist in a complex and heterodimerization modulates signaling pathways associated with pain and withdrawal.

## Materials and Methods

### Immunohistochemistry

30 µm thick free floating brain and spinal cord sections were collected in Tris-buffer saline (TBS) and processed for immunocytochemistry as described earlier [40]. Sections were incubated in 5% normal goat serum (NGS) for 1 h followed by incubation with a mixture of SSTR4 antibody (1∶400) and δOR antibody (1∶250) (Santa Cruz Biotechnology, Santa Cruz, CA) for 16 h at 4 °C. Following three subsequent washes with TBS, sections were incubated with goat anti-rabbit (Alexa-488) and donkey anti-goat (Alexa-594) secondary antibodies (Invitrogen, Burlington, ON) for 1 h to visualize SSTR4 and δOR respectively. Sections were mounted and viewed under Leica Confocal microscope. All the Figure composites were constructed using Adobe Photoshop (San Jose, CA) and NIH, ImageJ software. The protocols regarding animal care were followed in compliance with the Institute of Laboratory Animal Resources, Commission on Life Sciences, National Research Council and the University of British Columbia committee on Animal Care. The use of animals for the present study was approved by the University of British Columbia committee on Animal Care (Protocol # A06-0419).

### Receptor constructs and cell lines

cMyc-δOR in pCDNA3.1^+^/Hygro vector (hygromycin resistance) was purchased from TOP Gene Technologies (Montreal, Canada). Construct of HA-SSTR4 was made by using the pCDNA3.1^+^/Neo (neomycin resistance) as previously described [41], [42]. Stable transfections of HEK-293 cells expressing HA-SSTR4 and/or cMyc-δOR were prepared by transfection reagent (Invitrogen, Burlington, ON) as described earlier [41], [42].

### Co-immunoprecipitation and western blot analysis

Co-immunoprecipitation (Co-IP) in tissue extract prepared from brain regions and cell lysate prepared from HEK-293 cells was accomplished as described previously [43], [44]. Briefly, the brain tissue was homogenized in homogenization buffer and 250 µg of tissue protein was solubilized in 1 ml binding buffer (50 mM HEPES, 2 mM CaCl_2_, 5 mM MgCl_2_, pH 7.5) followed by treatment with SSTR4 specific agonist L-803087 (10 nM), δOR specific agonist SB-205607 (10 nM) alone or in combination for 30 min at 37°C. The lysates were than incubated with SSTR4 specific antibody (1:250) overnight at 4°C followed by 2h incubation with protein A/G agarose beads (Calbiochem, EMD Biosciences, Darmstadt, Germany). The purified samples were fractionated by electrophoresis and transferred to PVDF membrane. Blocking of the membrane, incubation with δOR specific primary and secondary antibodies and detection by chemiluminescence were performed following ECL Western blotting detection kit as per manufacturer's instructions (GE Healthcare, Piscataway, NJ). Membranes were developed using an Alpha Innotech FluorChem 8800 (Alpha Innotech Co., San Leandro, CA) gel box imager. Co-IP in cotransfected HEK-293 cells treated with receptor specific agonist alone or in combination was performed following similar steps. Cell lysates were immunoprecipitated with anti-cMyc antibody (1:500) and blotted with anti-HA specific antibody (1∶500).

Western blot for signaling pathways was performed by using phospho-and total specific antibodies to detect extracellular signal-regulated kinases (ERK1/2 and 5), Phospho-inositide 3-kinase (PI3K), protein kinase B (AKT) (Cell Signaling Technology, Danvers, MA) and protein kinase A (PKA) (Santa Cruz Biotechnology, Santa Cruz, CA). Whole cell lysates prepared from control and treated cells were quantified by Bradford assay, fractionated by electrophoresis, transferred to PVDF membrane and blotted using antibodies following standard protocol as described earlier. Densitometry for quantification was done using FluorChem software (Alpha Innotech) [42], [45].

### Microscopic Photobleaching-fluorescence resonance energy transfer (Pb-FRET) analysis in mammalian cells and cultured striatal neurons

HEK-293 cells expressing cMyc-δOR/HA-SSTR4 were treated with SST-14 (1 µM), L-803087 (10 nM) and SB-205607 (10 nM) alone and in combination for 15 min at 37°C. Post treatment, cells were fixed and processed for immunofluorescence immunocytochemistry using monoclonal anti-HA and polyclonal anti-cMyc primary antibodies followed with FITC and Cy3 conjugated secondary antibodies to create donor and acceptor pair (Sigma-Aldrich, Inc., St. Louis, MO). Pb-FRET analysis was performed as described earlier [42], [45]. The plasma membrane region was used to analyze the photobleaching decay on a pixel-by-pixel basis and FRET efficiency (E) was calculated [42], [45].

Primary culture of striatal neurons was prepared from 15 days old rat embryonic brains as described earlier [46]. Pb-FRET analysis performed in neuronal culture was exactly similar, except the receptor expression was determined by using SSTR4 and δOR specific antibodies followed with FITC and Cy3 conjugated secondary antibodies to create donor and acceptor pair and processed for Pb-FRET analysis.

### Receptor internalization

To study receptor internalization, HEK-293 cells stably transfected with cMyc-δOR/HA-SSTR4 were treated with SST-14 (1 µM), SB-205607 (10 nM) alone or in combination for 15 min at 37°C. Treatment was terminated by washing with ice cold Dulbecco’s PBS (GIBCO, Invitrogen, Burlington, ON, Canada). Cells were then fixed with 4% paraformaldehyde for 20 min on ice. To visualize intracellular expression of the receptor, cells were permeabilized with 0.2% Triton X-100 for 10 min and processed for immunocytochemistry as earlier reported [42], [45]. Merged images showing colocalization were generated by using NIH, ImageJ software and the photograph composites were made by using Adobe Photoshop (San Jose, CA). Quantification of immunofluorescence intensity was performed by using NIH, ImageJ software.

### Receptor coupling to adenylyl cyclase (AC)

Mono-and cotransfected HEK-293 cells expressing cMyc-δOR and/or HA-SSTR4 were processed for cyclic adenosine monophosphate (cAMP) assay as described [42]. Briefly, cells were treated with receptor specific agonists alone or in combination in presence of 20 µM forskolin (FSK) and 0.5 mM 3-isobutyl-1-methylxanthine (IBMX) for 30 min at 37°C. Cells were then collected in 0.1 N HCl and cAMP was determined by immunoassay using a cAMP Kit according to the manufacturer’s guidelines (BioVision, Inc., CA, USA) [42]. To determine concentration dependent effects on cAMP inhibition, cotransfected cells were treated with varying concentration of SST and δOR agonist alone or in combination and processed for cAMP assay.

### Statistical analysis

The data presented in this study were analyzed using GraphPad Prism 4.0. The data were statistically analyzed using one-way ANOVA and the post hoc Dunnett's test applied according to the experimental conditions to compare with treatments. Significant statistical differences were taken at *p<0.05. Results are presented as mean ± SD unless otherwise stated.

## Results

### δOR colocalizes with SSTR4 in rat brain cortex, striatum and spinal cord

**Figure 1:**
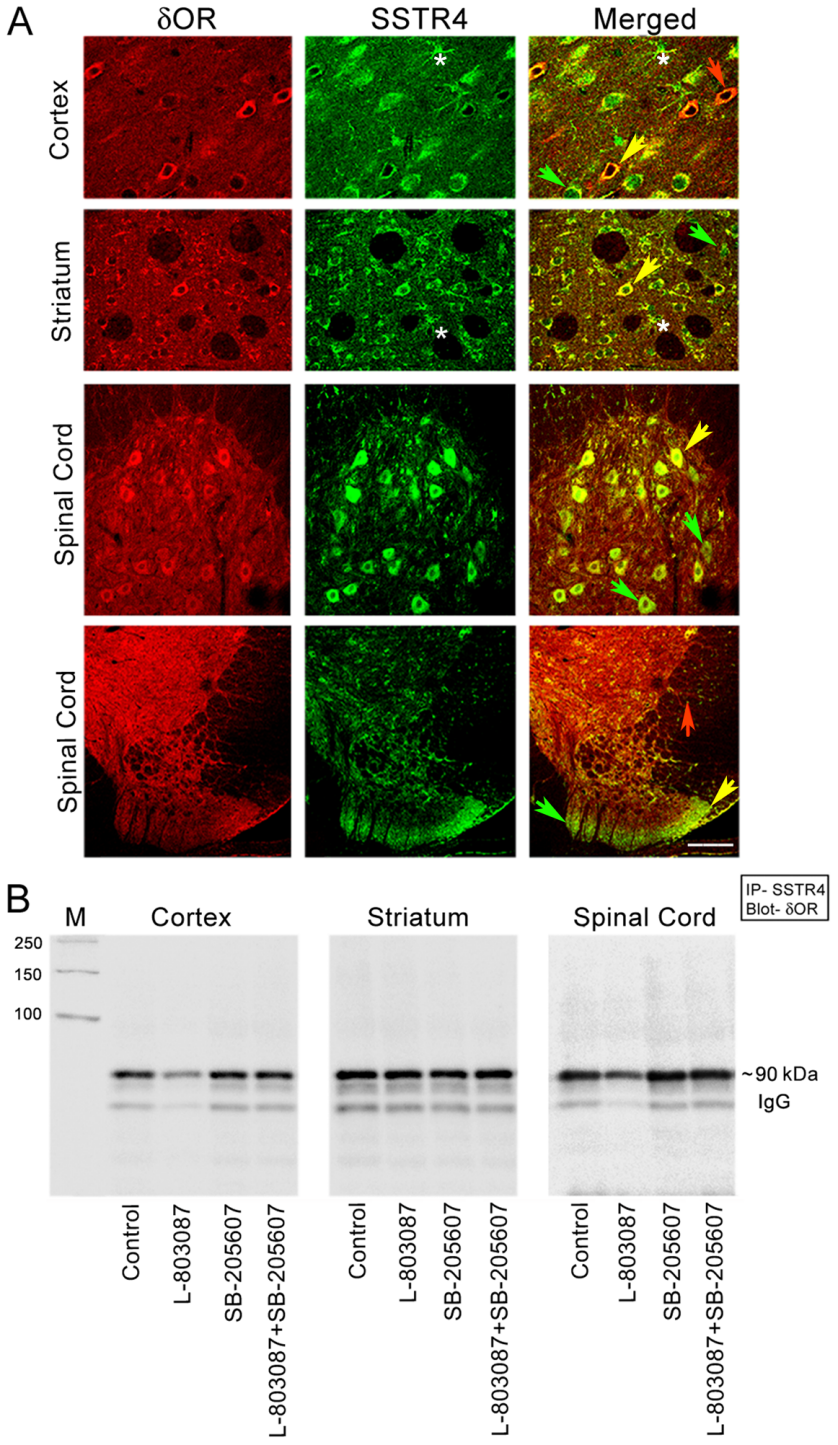
Colocalization and Co-Immunoprecipitation of SSTR4 and δOR in rat brain and spinal cord. (**A**) Representative confocal photomicrographs illustrating the colocalization of SSTR4 and δOR in rat brain cerebral cortex, striatum and spinal cord. 30 µm sections from brain and spinal cord were incubated with SSTR4 and δOR specific primary antibodies, followed by incubation with secondary antibodies. The expression of δOR (red), SSTR4 (green) and colocalization is identified in orange yellow color. Note, all δOR positive neurons colocalized with SSTR4 in the three brain regions including cortex, striatum and ventral and dorsal horn of spinal cord respectively. In addition, SSTR4 positive neurons devoid of colocalization are indicated by green arrows in respective panel. In spinal cord, nerve fibres positive to δOR were devoid of colocalization with SSTR4 (red arrows spinal cord bottom panel). Astrics (*) indicate astrocytes in cortical and striatal brain sections. (Scale bar  =  10 µm for upper panels and 100 µm for bottom panel; n  =  3). (**B**) Expression of δOR in SSTR4 immunoprecipitate prepared from rat brain and spinal cord. Membrane preparation from cortex, striatum and spinal cord tissue lysate was treated with SSTR4 specific agonist (L-803087), δOR specific agonist (SB-205607) alone or in combination for 30 min at 37°C. Following treatments, tissue lysate was immunoprecipitated with SSTR4 antibody and immunoblotted for δOR specific antibody as described in Methods section. The expression of δOR at approximately ∼90 kDa indicates that δOR and SSTR4 exist in a complex in brain.

We first determined whether SSTR4 and δOR colocalize in brain region associated with different functions. Accordingly, colocalization of SSTR4 and δOR was determined in three different regions including the cortex, striatum and the spinal cord (**Figure 1A** **)**. In cortical brain regions δOR and SSTR4-like immunoreactivity was selectively expressed in specific neuronal populations, displaying distinct morphology and a variable degree of colocalization. Three different neuronal populations, either positive to SSTR4 (green arrow), δOR (red arrow) and displaying colocalization (yellow arrow) were identified. In the cortex, putative glial cells (morphological identification) positive to SSTR4 were lacking δOR-like immuno-reactivity and were devoid of colocalization. In the striatum, two different neuronal populations, either positive to SSTR4 (green arrow) and displaying colocalization (yellow arrow) were observed. Unlike cortex, in the striatum, putative glial cells were strongly positive to δOR and displayed colocalization with SSTR4 (shown by *). In the spinal cord, SSTR4 and δOR were strongly expressed in ventral and dorsal horn, and displayed a region specific colocalization. Motor neurons in the ventral horn expressed strong SSTR4-like immunoreactivity in comparison to δOR. We observed that all δOR positive neurons colocalized with SSTR4 indicating that δOR expression was limited to SSTR4 positive neurons. In addition, some neurons were devoid of colocalization but expressed only SSTR4. Strongly positive δOR cell bodies in the dorsal horn (substantia gelatinosa) displayed a selective colocalization. In comparison, densely innervated nerve fibers in spinal cord rich in δOR immunoreactivity were lacking colocalization. These results provide first molecular basis for pharmacological and physiological interactions between these two receptor subtypes. The specificity of immunoreactivity was determined in the absence of primary antibodies and in presence of pre-immune serum or antigen adsorbed antibodies as described previously [40]. Taken together, consistent observations in three different brain regions revealed that all δOR positive neurons coexpress SSTR4, whereas, some neurons positive to SSTR4 were devoid of δOR like immunoreactivity.

### Expression of δOR in SSTR4 immunoprecipitate prepared from rat brain cortex, striatum and spinal cord

In support of colocalization studies, Co-IP assay was performed to determine the δOR expression in SSTR4 immunoprecipitate. δOR was detected in SSTR4 immunoprecipitate prepared from cortical, striatal and spinal cord at the expected molecular size of ∼90 kDa in basal as well as following treatment with receptor specific agonists (**Figure 1B**). Since no loading control is available for quantification in immunoprecipitate assay, the low expression of δOR seen in cortex and spinal cord upon treatment with SSTR4 agonist is not conclusive. These results support that SSTR4 and δOR exist in a heteromeric complex in CNS.

### Constitutive heteromeric complex between δOR and SSTR4 in cultured striatal neuronal cells

**Figure 2:**
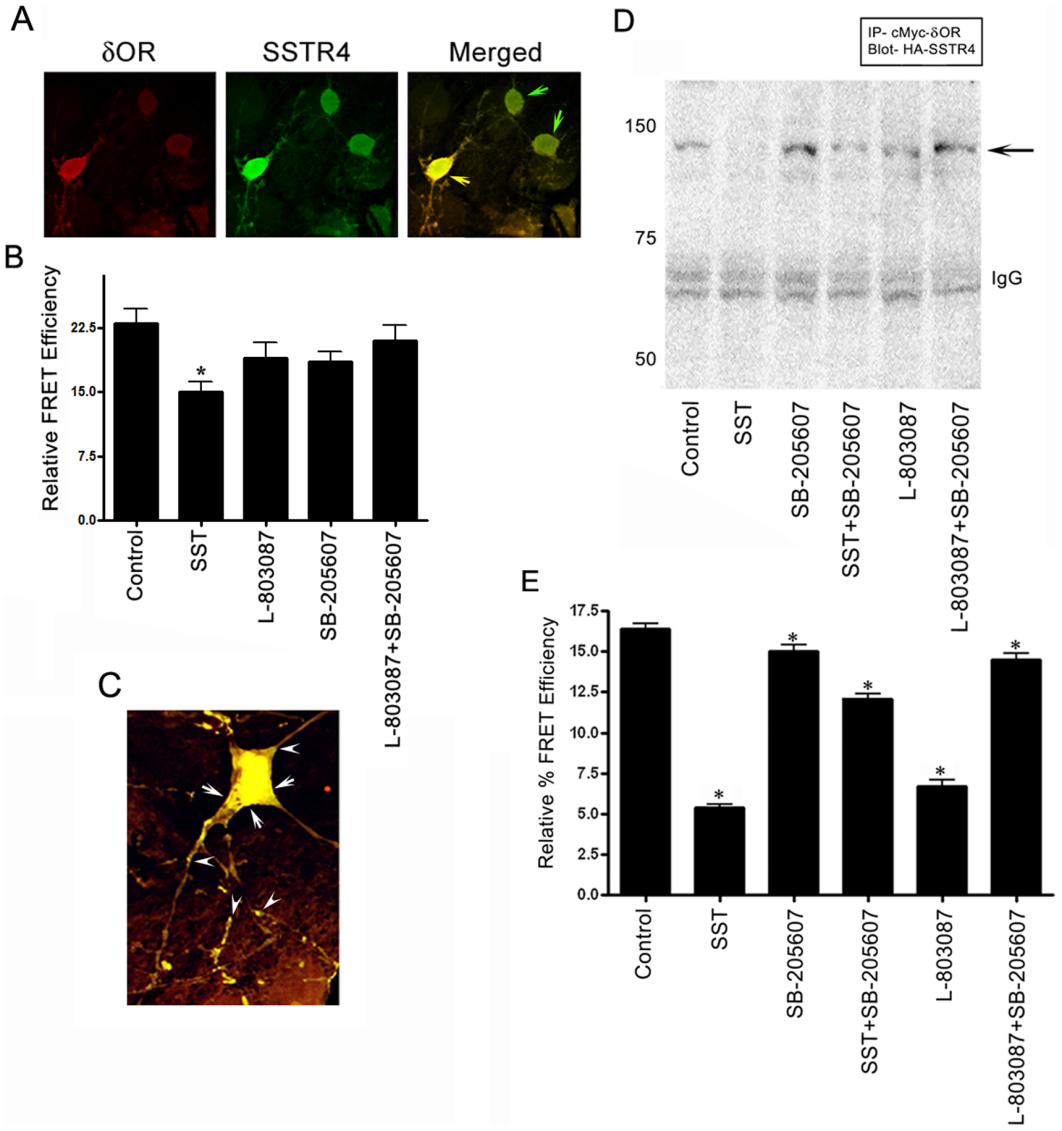
SSTR4 and δOR heterodimerization in cultured striatal neurons. (**A**) Confocal photomicrographs displaying receptor specific colocalization of SSTR4 and δOR in cultured striatal neurons. Note the neuronal population devoid of colocalization (green arrows). (**B**) Histogram displaying relative effective FRET efficiency in control and neurons treated with SST, L-803087 and SB-205607 alone or in combination. Neurons lacking colocalization (green arrows Panel A) were used for specificity. (**C**) Representative single neuronal cell used to determine FRET efficiency on soma (areas indicated by arrows) as well as proximal ending and neuronal processes (identified by arrowheads). FRET efficiency from area indicated by arrowheads was significantly lower than the areas indicated by arrows representing heterogeneous heterodimerization in neuronal cells. (**D**) Co-immunoprecipitation analysis illustrating the expression of SSTR4 in δOR immunoprecipitate prepared from control and treated cotransfected HEK-293 cells. Cells were treated with SST (1 µM), L-803087 and SB-205607 (10nM each) alone or in combination. Post treatment, cell lysate prepared was immunoprecipitated with anti-cMyc antibody (δOR) and immunoblotted with anti-HA antibody to detect SSTR4 expression. Note the loss of receptor heterodimerization upon treatment with SST or SSTR4 specific agonist in comparison to control, whereas receptor complex was stabilized in presence of δOR agonist (SB-205607) alone or in combination with SSTR4 specific agonist L-803087. (**E**) Histogram illustrating relative FRET efficiencies in cotransfected HEK-293 cells. SSTR4/δOR exists as preformed heterodimers in basal condition. Note the loss in relative FRET efficiency in presence of SST or SSTR4 specific agonist. Conversely, SB-205607 alone or in combination with SST or L-803087 enhanced relative FRET efficiency. Data analysis was done by using ANOVA and *post hoc Dunnett’s* test to compare with control and treated conditions. (*, *p*<0.05; 50–60 cells were analyzed from three different experiments).

Colocalization and Co-IP in cortical and striatal neurons can be taken in account to support interaction between SSTR4 and δOR in brain, however, heterodimerization is sensitive to distance, conformational dynamic and receptor orientation at the surface. We next determined the presence of heteromeric complex formation directly in striatal cultured neurons by Pb-FRET analysis. FRET signals were obtained from bleaching profile of donor molecules in presence or absence of acceptor molecules identified by using FITC- and Cy3- labeled antibodies. δOR and SSTR4 displayed colocalization in neuronal cells in a heterogeneous manner throughout the cell body with sparsely distributed colocalization in neuronal processes **(** **Figure 2A** **)**. As illustrated in **Figure 2B**, high relative FRET efficiency of 23±3% in basal condition indicated a constitutive heterodimerization between δOR and SSTR4 in striatal neurons. In neuronal cells, the relative FRET efficiency of 15±2%, 19±3% and 18.5±2% was observed upon treatment with SST (1 µM), L-803087 and SB-205607 (10 nM each) respectively. Of note, simultaneous activation of SSTR4 and δOR displayed FRET efficiency of 21±3% which was relatively higher than the single receptor agonist treatment but comparatively less than the basal condition. As demonstrated in **Figure 2C**, the relative FRET efficiency was significantly variable at the neuronal cell body confirming heterogeneous population of heterodimers in neuronal cells. The relative FRET efficiency obtained from neuronal processes indicated by arrowheads was significantly lower despite receptor colocalization in comparison to the regions identified by arrows on neuronal cell bodies (**Figure 2C**). These results provide direct evidence for the association of δOR and SSTR4 as heterodimers in the native system. The loss of effective FRET efficiency in neuronal cells lacking colocalization supports the specificity and selectivity of receptor heterodimerization (**Figure 2A** **; green arrows**).

### Interaction between δOR and SSTR4 in mammalian HEK-293 cells

Colocalization and co-immunoprecipitation studies in the rat brain cortex, striatum and spinal cord along with the Pb-FRET analysis in the neuronal cells indicated the formation of heteromeric complex between SSTR4 and δOR in native system. To overcome the complexity involved in examining native systems expressing multiple receptors in a single neuronal cell and brain slices, we next determined physical interaction between SSTR4 and δOR in stably cotransfected mammalian HEK-293 cells using Co-IP and Pb-FRET analysis. The membrane extract prepared from control and cells treated with SST (1 µM), L-803087 and SB-205607 (10 nM) alone or in combination was immunoprecipitated with cMyc- antibody (δOR) and immunoblotted with HA- antibody to detect SSTR4. Like brain regions, HA-SSTR4 was detected in the δOR immunoprecipitate at the expected size of SSTR4/δOR heteromeric complex (∼90 kDa) in control and in cells treated with the receptor specific agonists (**Figure 2D** **)**. Importantly, the expression of SSTR4 in δOR immunoprecipitate was significantly diminished upon treatment with SST or SSTR4 specific agonist. Conversely, complex formation was stabilized in presence of SB-205607 alone and in combination with SSTR4 specific agonist L-803087. Immunoprecipitate of cotransfected cells prepared in absence of δOR or SSTR4 specific primary antibody was devoid of SSTR4 or δOR expression respectively **(Figure S1)**. These observations confirm the specificity of the heterodimerization between SSTR4 and δOR in cotransfected cells comparable to *in vivo* system.

### Receptor heterodimerization at cell surface is predominantly regulated by δOR

We next determined microscopic Pb-FRET analysis to demonstrate receptor heterodimerization at the cell surface in cotransfected HEK-293 cells **(** **Figures 2E**, **S2 and S3)**. As illustrated in **Figure 2E**, Pb-FRET analysis showed a relatively high FRET efficiency of 16.40±0.54% in the basal condition indicating constitutive heteromeric complex of SSTR4/δOR. Upon treatment with SST or L-803087, effective FRET efficiency was significantly decreased to 5.40±0.27% and 6.69±0.65% respectively. In contrast, relative FRET efficiency of 15±0.7% was observed upon treatment with δOR (SB-205607) which was comparable to control. Furthermore, simultaneous activation of δOR and SSTR4 with SB-205607 and SST or L-803087 resulted in relative FRET efficiency of 12.1±0.5% and 14.51±0.71%, respectively **(** **Figures 2E** **and** **S3)**. The relative FRET efficiency remained comparable irrespective of either SSTR4 or δOR being used as donor molecule in bleaching experiment. These results attest to the findings made in brain slices and neuronal cells and indicate the agonist dependent regulation of δOR and SSTR4 heterodimerization process. Furthermore, consistent with previous observations our results also indicate that activation of single interacting receptor is sufficient enough to trigger association or dissociation of heterodimers.

### SSTR4 and δOR heterodimerization alter receptor specific trafficking properties upon coactivation in HEK-293 cells

**Figure 3:**
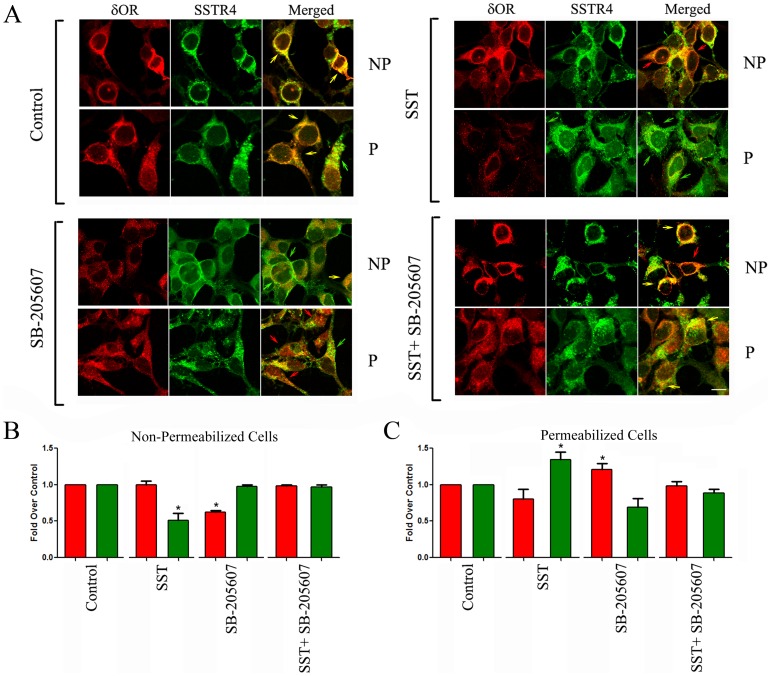
Co-activation of SSTR4 and δOR retained receptors expression at the cell surface. (**A**) Representative confocal photomicrographs illustrating membrane and intracellular expression of SSTR4 and δOR in non-permeabilized (NP) and permeabilized (P) HEK-293 cells. Indicated color in red, green and yellow/orange (merged images) represents the expression of δOR, SSTR4 and colocalization respectively. In cotransfectants, the activation of SSTR4 (upper panel right) or δOR (lower panel left) preferentially promotes receptor internalization which was blocked upon combined agonist treatment. Histogram in panels **B and C** showed the quantification of receptor expression in non-permeabilized (NP) and permeabilized (P) conditions respectively performed by using NIH Image J software. Note the significant loss in expression of δOR (B, red histogram) and SSTR4 (B, green histogram) at cell surface upon treatment with receptor specific agonists as indicated. Intracellular expression of δOR (C, red histogram) and SSTR4 (C, green histogram) was significantly enhanced following treatments with SB-205607 and SST alone without having any discernible effects in combination (*, *p*<0.05). Scale bar  =  10 µm.

SSTR4 or δOR like many other GPCRs exerts distinct role on signaling pathways when expressed at the cell surface or intracellularly. We next monitored and quantified SSTR4 and δOR membrane and intracellular expression in HEK-293 cells stably transfected with cMyc-δOR and HA-SSTR4 following treatment with receptor-specific agonists. Like neuronal cells, in basal condition SSTR4 and δOR are well expressed and displayed strong colocalization at the cell membrane as well as intracellularly (**Figure 3A**). Upon treatment with δOR specific agonist SB-205607, δOR expression was reduced at the membrane, whereas, cells exhibited strong expression of SSTR4 at membrane comparable to control **(** **Figure 3A and B** **)**. Agonist induced δOR Internalization resulted in weak colocalization with SSTR4 at the cell surface, accompanied with strong cytoplasmic colocalization of these two receptors. Importantly, permeabilized cells displayed two different populations intracellularly either colocalized or δOR **(** **Figure 3A and C** **)**. Like δOR, the expression of SSTR4 at the cell surface was significantly diminished in the presence of SST and, consequently, enhanced intracellularly when compared to control **(** **Figure 3A, B and C** **)**. Interestingly, unlike the individual receptor activation, co-activation of SSTR4 and δOR in combination displayed colocalization at cell surface as well as intracellularly, without any discernible changes from control **(** **Figure 3A, B and C** **)**. The present data demonstrates that receptor internalization induced by a single treatment is opposed by simultaneous activation of both receptors. Most importantly, these results elucidate that the unique property of individual receptors was changed or stabilized in cotransfected cells upon activation of both protomers, attributed to receptor heterodimerization. These results also strengthen the concept that SSTR4 mediated retention of δOR at the cell surface might enhance analgesic effect.

### Changes in receptor coupling to AC

**Figure 4:**
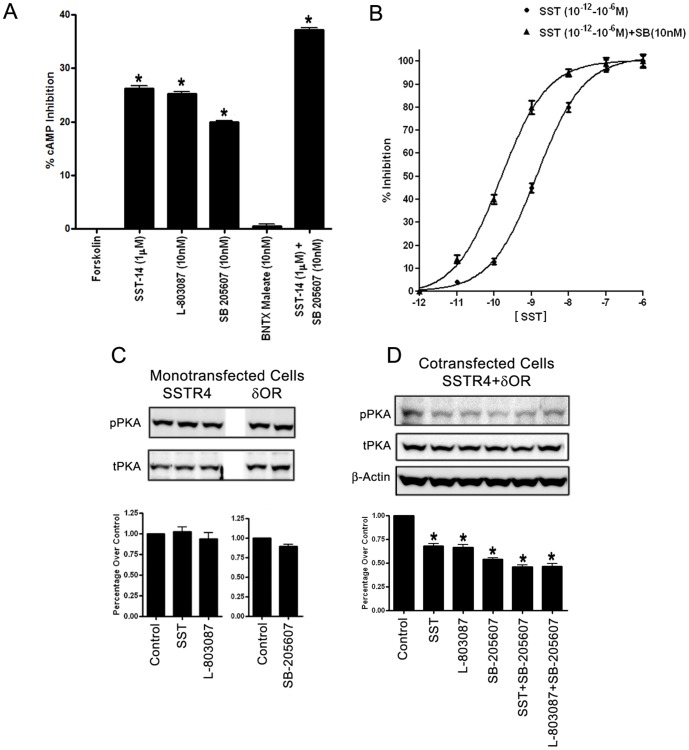
Regulation of cAMP/PKA signaling pathways in receptor and agonist dependent manner. (A) Receptor coupling to adenylyl cyclase. SST, L-803087 and SB-205607 displayed significant inhibition of FSK stimulated cAMP in comparison to control. Data is representative of three independent experiments and presented as % inhibition upon treatment as indicated. (B) Concentration dependent inhibition of cAMP in HEK-293 cells. Treatment of cells with FSK alone was taken as 0% inhibition and treatment with forskolin and SST (1 µM) was considered as 100% inhibition. Note the significant increase in the efficiency of cAMP inhibition upon treatment with SST (10^−12^–10^−6^ M) in combination with SB-205607 (10 nM). (C and D) PKA phosphorylation in mono-and/or cotransfected cells. HEK-293 cells expressing SSTR4 and/or δOR were treated for 15 min at 37°C as indicated and cell lysate prepared was subjected to western blot analysis. In monotransfected cells, the status of phospho-PKA was comparable to basal upon receptor specific activation (C). In cotransfected cells, significant inhibition of PKA phosphorylation was observed which was further enhanced upon combined agonist treatment as indicated (D). Densitometric analysis for phospho-PKA was performed by using β-actin or total as loading control and data analysis was done by using ANOVA and *post hoc* Dunnett’s to compare against basal level (*, *p*<0.05).

Receptor coupling to AC and the formation of cAMP through different heterotrimeric G-proteins is a characteristic of GPCRs functionality [47]. SSTR and OR subtypes negatively couple to AC and inhibit the formation of cAMP in a receptor-specific Gi dependent manner. To test whether activation of SSTR4 or δOR alone or in combination regulates cAMP in a distinct manner, cotransfected HEK-293 cells were treated with FSK with and without SST, SSTR4 and δOR specific agonists for 30 min and processed for cAMP. As shown in **Figure 4A**, FSK stimulated cAMP was inhibited by 26.2±0.85, 25.2±0.64 and 19.9±0.63% in the presence of SST, L-803087 and SB-205607, respectively. SST-14 (1 µM) in combination with SB-205607 (10 nM) induced an inhibition by 37.2±0.56% which was significantly higher than the single agonist treatment. Having established that SST, L-803087 and SB-205607 inhibit cAMP formation with enhanced inhibition in combination prompted us to determine the concentration dependent effect of SST (10^−12^ – 10^−6^ M) alone or in combination with SB-205607 (10^−8^ M) on cAMP inhibition **(** **Figure 4B** **)**. The treatment of cells with increasing concentration of SST (10^−12^ – 10^−6^ M) exhibit enhanced inhibition of cAMP in dose dependent manner. Interestingly, the inhibition of cAMP was significantly enhanced upon treatment with SST (10^−12^–10^−6^ M) in combination with SB-205607 (10 nM) in comparison to SST (1 µM) treatment alone **(** **Figure 4B** **)**. In contrast, increasing concentrations of SB-205607 (10^−13^–10^−8^ M) alone or in combination with SST (1 µM) also displayed cAMP inhibition but the efficiency of inhibition was significantly lower than SST (10^−12^–10^−6^ M) alone or in combination with SB-205607 (10 nM) (Data not shown). Taken in consideration, these data strongly indicate that maximal inhibition achievable was significantly higher upon co-activation of the receptors in comparison to single receptor activation. Also, indicates that SSTR4 and δOR function synergistically and activation of SSTR4 in heteromeric complex is predominantly responsible for inhibition of FSK stimulated second messenger cAMP.

### Negative regulation of cAMP formation is associated with the inhibition of PKA phosphorylation

Dolan et al, proposed a biphasic modulation of nociception and demonstrated the role of cAMP/PKA pathway in hypoalgesia and hyperalgesia [48]. To determine whether the inhibition of FSK stimulated cAMP also regulates PKA phosphorylation, mono and/or cotransfected cells were treated with receptor specific agonist alone and/or in combination for 15 min at 37 °C and cell lysate processed for PKA expression and phosphorylation. Upon treatment with receptor specific agonists in SSTR4 or δOR monotransfected cells, the status of phosphorylated PKA remained comparable to the control (**Figure 4C** **)**. In comparison, PKA phosphorylation was inhibited in presence of SST, SSTR4 and δOR agonist in cotransfectants and cells displayed significantly higher inhibition of phospho-PKA upon combined treatment as indicated (**Figure 4D** **)**. Furthermore, inhibition of PKA phosphorylation correlates with the suppression of FSK stimulated cAMP and strengthens the notion that cAMP/PKA function in an integrated manner. To demonstrate G protein dependency, we next determined the effect of Gi inhibitor PTX on PKA phosphorylation. Cotransfected cells pretreated with PTX displayed significantly high level of PKA phosphorylation in control or upon agonist specific treatments **(Figure S4A)**. Taken together, these findings demonstrate a close association between cAMP/PKA, SSTR4/δOR heterodimerization and Gi dependency.

### Receptor heterodimerization modulates ERK1/2 and ERK5 MAPKs signaling

**Figure 5:**
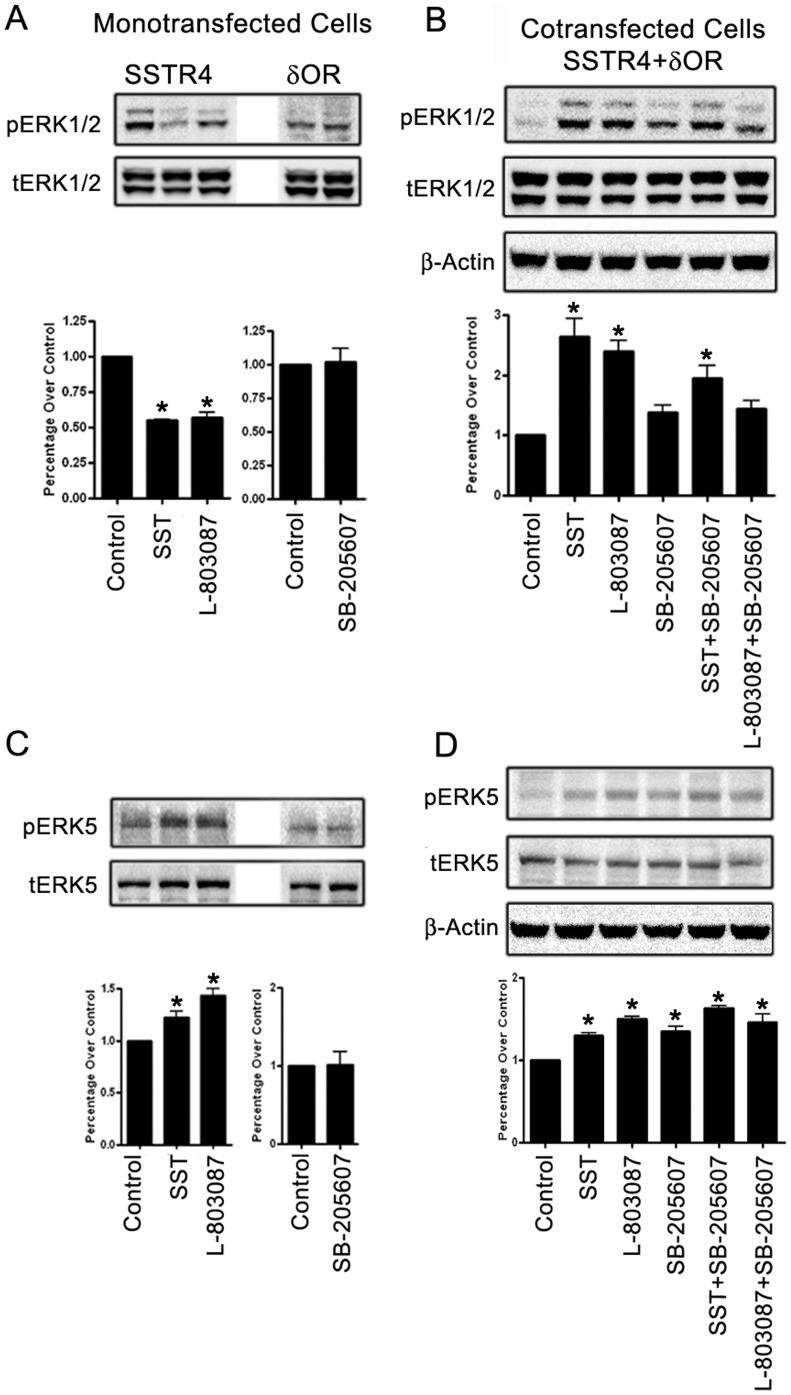
Receptors mediated changes in ERK1/2 and ERK5 phosphorylation. (**A**) HEK-293 cells expressing SSTR4 and /or δOR were treated as indicated for 15 min at 37°C and cell lysate was subjected to Western blot analysis. In SSTR4 monotransfected cells, SST and SSTR4 agonist resulted in inhibition of phospho-ERK1/2 when compared to control, whereas, δOR activation was without any significant effect on ERK1/2 phosphorylation. (**B**) In cotransfected cells, SSTR4 activation displayed increased ERK1/2 phosphorylation. In presence of δOR agonist alone or in combination with SST or L-803087, ERK1/2 activation was decreased although remained significantly higher than control. (**C**) In monotransfected cells, SSTR4 activation resulted in enhanced phospho-ERK5 expression, whereas, no change in the phospho-ERK5 levels was observed upon δOR activation. (**D**) In cotransfected cells, increased ERK5 phosphorylation was observed upon SSTR4 or δOR activation alone or in combination. Note that the combined agonist treatment had pronounced effect on ERK5 phosphorylation. Densitometric analysis for the blots (in Panels A-D) was performed by using β-actin or total as loading control and data analysis was done by using ANOVA and *post hoc* Dunnett’s to compare against basal level (*, *p*<0.05).

Receptor oligomerization exerts crucial role in the modulation of multiple downstream signaling pathways with distinct physiological responses of cells. MAPKs, including ERKs, are linked to the induction and maintenance of neuropathic pain [49], accordingly, the status of ERK1/2 phosphorylation was determined in mono-and/or cotransfected cells. Monotransfected cells expressing SSTR4 displayed inhibition of phospho-ERK1/2 in presence of SST and L-803087 in comparison to control **(** **Figure 5A** **)**. In contrast, in cells expressing δOR, the status of phospho-ERK1/2 was comparable to the control in the presence of δOR agonist SB-205607 and interestingly cells were devoid of phospho-ERK1 isoform. In cotransfected cells, ERK1/2 phosphorylation was comparatively higher upon SSTR4 activation than δOR when compared to the control **(** **Figure 5B** **)**. SST or SSTR4 specific agonist in combination with SB-205607 displayed enhanced ERK1/2 phosphorylation although relatively less in comparison to SST or SSTR4 agonist treatment alone. Our results further revealed significant differences in the phosphorylation of ERK1 in comparison to ERK2 isoform. To ascertain whether ERK1/2 is Gi sensitive or insensitive, cells were exposed to PTX prior to agonist treatment. As shown, phospho-ERK1/2 was highly expressed in basal condition without any significant changes in cells treated with receptor specific agonist **(Figure S4B)**. These results are an indication of Gi dependency in cotransfected cells.

Pain hypersensitivity in response to nerve injury has been linked to the activation of ERK5 [50]. To determine whether crosstalk between SSTR4 and δOR and changes in the status of ERK1/2 are also involved in the regulation of ERK5 phosphorylation, mono-and/or cotransfected cells were processed for total and phosphorylated ERK5 expression. Monotransfected cells expressing SSTR4 displayed increased phospho-ERK5 in the presence of SST and L-803087, whereas, the status of phospho-ERK5 remained comparable with or without agonist treatment in cells expressing δOR **(** **Figure 5C** **)**. In comparison, cells cotransfected with SSTR4/δOR, SSTR4 mediated activation of ERK5 was maintained with or without SB-205607 **(** **Figure 5D** **)**. Interestingly, ERK5 phosphorylation was completely abolished in cells pre-treated with PTX (**Figure S4C)**. These results uncovered Gi dependent ERK5 phosphorylation and signify that SSTR4 exerts differential effects on the regulation of ERK1/2 and ERK5 phosphorylation in mono-and/or cotransfected cells.

### PI3K phosphorylation is receptor specific and dependent on Gi

**Figure 6:**
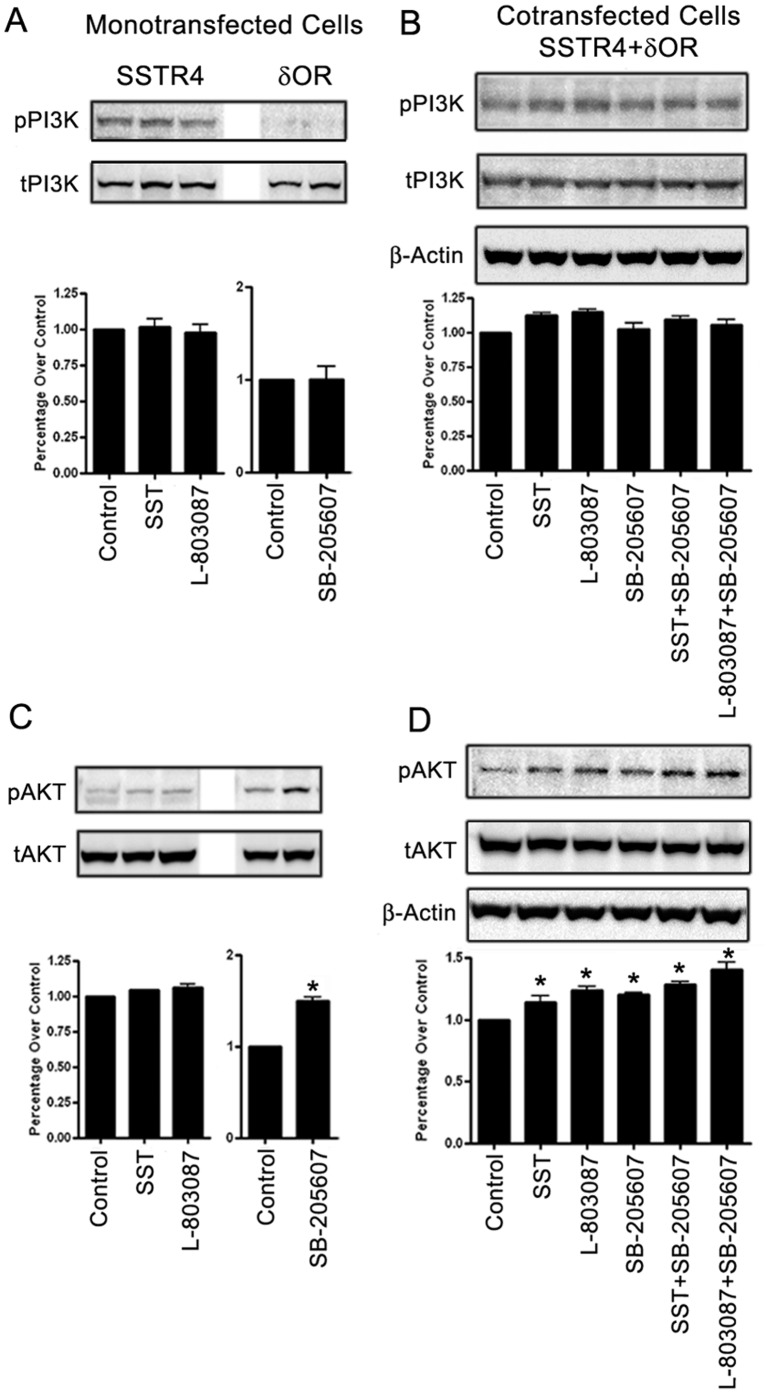
Changes in AKT phosphorylation are independent to PI3K activation. (**A**) HEK-293 cells expressing SSTR4 and /or δOR were treated as indicated for 15 min at 37°C and cell lysate prepared post treatment was subjected to western blot analysis to detect phospho and total-PI3K and AKT expression. The status of phospho-PI3K remained comparable to basal upon treatment with SST or SSTR specific agonist in cells expressing SSTR4. In contrast, δOR monotransfectant were devoid of phospho-PI3K expression with or without receptor specific treatment. (**B**) Significant activation of phospho-PI3K was observed in cotransfected cells at the basal level which remained comparable upon agonist treatments as indicated. (**C**) δOR monotransfected cells displayed significant increase in phospho-AKT expression upon treatment with receptor specific agonist in comparison to control, whereas, no discernible changes in phospho-AKT were observed in SSTR4 monotransfected cells. (**D**) In cotransfected cells, SSTR4 activation with SST or L-803087 alone or in combination with SB-205607 significantly enhanced AKT phosphorylation, with pronounced effect upon combined agonist treatment. Histograms illustrate densitometry for the blots for respective panels using β-actin or total as loading control. Data analysis was done by using ANOVA and *post hoc* Dunnett’s to compare against basal level (*, *p*<0.05).

Previous studies have shown that the inhibition of PI3K abrogates the anti-nociceptive effects of µOR and δOR agonists [51]. We next compared the status of activated PI3K in cells expressing SSTR4 and/or δOR. As shown in **Figure 6A**, the status of activated PI3K was comparable to control in monotransfectant expressing SSTR4 upon treatment with SST or SSTR4 specific agonist. In contrast, phospho-PI3K expression was not detected in cells expressing δOR with or without agonist specific activation. In cotransfected cells, sustained activation of phospho-PI3K was observed in control as well as following treatment with SSTR4 or δOR specific agonist alone and in combination **(** **Figure 6B** **)**. These results indicate that PI3K phosphorylation in cotransfected cells is predominantly SSTR4 dependent and comparable to SSTR4 monotransfected cells. Activation of PI3K was abolished in cells treated with PTX indicating the Gi dependent effect on PI3K phosphorylation following either receptor activation **(Figure S4D)**.

### Changes in phospho-AKT as downstream effectors of PI3K are not associated with PI3K

The PI3K/AKT signaling pathway is linked to the development of neuropathic pain [52]. Whether PI3K and its downstream effector AKT function in a coordinated manner or distinctly, we next examined the status of AKT phosphorylation in cells expressing SSTR4 and/or δOR upon treatments as indicated **(** **Figure 6C and D** **)**. In SSTR4 monotransfected cells, activated AKT was comparable to control upon treatment with SST and SSTR4 specific agonist (L-803087) **(** **Figure 6C** **)**. In contrast, cells expressing δOR exhibit enhanced AKT phosphorylation in presence of SB-205607 when compared to control. These results are contrary to the pattern of PI3K expression in δOR monotransfectant. In cotransfected cells, consistent expression of phospho-AKT was observed upon treatments as indicated in comparison to control. Interestingly, SST or L-803087 had stimulatory effect on AKT phosphorylation in combination with SB-205607 **(** **Figure 6D** **)**. Inhibition of Gi protein in presence of PTX resulted in an increased AKT activation, indicating AKT phosphorylation is Gi independent **(Figure S4E)**. Taken together, these results indicate that AKT can also be activated independent of PI3K signaling pathways in mono-and cotransfected cells. Moreover, it should be noted that in cotransfected cells phospho-PI3K while regulated by SSTR4, AKT phosphorylation is predominantly under the influence of δOR.

## Discussion

In the present study, we provide morphological, biochemical, and biophysical evidences supporting oligomerization between δOR and SSTR4 *ex vivo* and *in vitro* in rat brain, cultured striatal neurons and stably transfected HEK-293 cells. These observations suggest that heterodimerization of δOR and SSTR4 in fact creates a novel receptor complex with functional diversity. The consequences of cellular response upon activation of both interacting protomer are more pronounced than a single receptor in heterologous system. We also describe the heterogeneous complex formation between δOR and SSTR4 in neuronal cells despite strong receptor colocalization. The activation of SSTR4 and δOR in combination leads to a greater inhibition of cAMP/PKA and modulation of ERK1/2 and ERK5 than the activation of individual receptors. Importantly, while cell surface heterodimerization is essentially regulated by δOR, the signaling pathways are predominantly influenced by SSTR4. Our observations have uncovered that MAPKs (ERK1/2 and ERK5) and PI3K/AKT phosphorylation is regulated in Gi dependent and independent manner in cotransfected cells. This is the first comprehensive description providing receptor interaction and its functional consequences in modulation of pain related signaling pathways.

Despite strong colocalization of SSTR4 and δOR in neuronal cell body and processes, differences in relative FRET efficiency imply that receptor orientation and conformational dynamics at the cell surface is crucial for protein-protein interaction. Since neuronal cells expressed more than one receptor subtypes thus, the possibility of complex formation with other receptors cannot be ruled out from the discussion. However, future studies are warranted to determine the functional significance of heterogeneity in the receptor complex formation at neuronal soma and neuronal processes in synaptic transmission and plasticity.

The loss of FRET efficiency upon activation of SSTR4 with either SST or receptor specific agonist is in agreement with the concept of receptor complex dissociation and internalization. Consistent with previous studies, we argue that activation of δOR preferentially distinguishes between homodimers of δOR from heteromeric complex of SSTR4 and δOR. In contrast to cotransfected HEK-293 cells, FRET efficiency in neuronal cells was relatively less when neuronal cells were treated with either SSTR4 or δOR agonist in comparison to control. Although, co-activation of both interacting protomers exhibit lower FRET efficiency, but was enhanced in comparison to SSTR4 activation alone. The selective and preferential heterodimerization in native and heterologous system ruled out the possibility of artifact in FRET due to receptor over expression. The membrane expression of ORs is critical for analgesic effect and studies have shown that µOR is more efficient in regulating nociception than δOR due to elevated membrane expression. In support, our results in cotransfected cells revealed that the presence of SSTR4 averts δOR internalization and retains the receptor at cell surface as a complex, whereas, activation of individual receptor displayed receptor trafficking like monotransfectants. These results indicate a close association between receptor trafficking and heterodimerization. Importantly, our results suggest that SSTR4 might enhance δOR mediated analgesic properties by retaining δOR at the cell surface.

cAMP pathway in G-protein dependant manner is associated with nociception [53]. Increased cAMP is allied with behavioural symptoms of withdrawal and enhanced action potential in neurons [54]. Moreover, inhibitors of PKA alleviate withdrawal symptoms [55]. The synergistic activation of SSTR4 and δOR display pronounced inhibition of cAMP formation in comparison to the activation of SSTR4 or δOR independently. Increased cAMP formation is seen as a consequence of withdrawal upon chronic use of morphine, thus, our results demonstrate that SST in combination with opioids might maintain sustained inhibition of cAMP even during withdrawal [54], [56]. Most importantly, significant inhibition of cAMP despite the loss in relative FRET efficiency upon activation of SSTR4 indicates that receptor coupling to AC and receptor heterodimerization are two independent processes. It is highly possible that this inhibition is attributed to SSTR4 homodimers. Previous studies have shown that PKA inhibitor H-89 reversed the mechanical hypoalgesia induced by cAMP analogue [48]. Our results demonstrate an inhibition of PKA phosphorylation upon SSTR4 and δOR heterodimerization and provide direct physiologically relevant evidence that SSTR4 in concert with δOR might involve in inhibition of cAMP/PKA. In addition, the present results suggest that the inhibition of cAMP/PKA is Gi dependent because PKA phosphorylation was significantly upregulated in the presence of Gi inhibitor PTX.

MAPKs play critical role in pain progression [57], [58] http://molpharm.aspetjournals.org/cgi/content/full/64/6/1317 - REF2#REF2http://molpharm.aspetjournals.org/cgi/content/full/64/6/1317 - REF3#REF3. Activated ERK has been shown in electrical stimulation of nociceptive afferents or peripheral nociceptors using capsaicin [59]. Also, the essential role played by ERK1/2 in counteracting morphine tolerance has also been demonstrated by using inhibitors of ERK1/2 [60]. Consistent with these observations, our results demonstrate that SSTR4 mediated activation of ERK1/2 may serve to assuage morphine tolerance upon prolonged drug administration. Interestingly, ERK1 ablated animals displayed enhanced constitutive ERK2 phosphorylation indicating predominant role of ERK2 in nociception [61]. Consistent with these observations, results described in the present study suggest that SSTR4/δOR activated ERK2 might play essential role in nociception. Interestingly, the molecular mechanism for the changes in ERK phosphorylation in response to SST in monotransfected cells in comparison to cotransfected cells warrants future studies. Furthermore, specificity towards opiate receptors and displacement of opiate binding by SST and its analogues as shown in earlier studies might also results in distinct regulation of ERK or other downstream signaling pathways in cotransfected cells [62]–[64]. Similarly, previous study has indicated possible binding of κOR selective opioid agonist ethylketocyclazocine with SSTRs and modulation of downstream signaling pathway in hepatocellular carcinoma (HepG2) cells [65]. HepG2 cells do not express SSTR1 and SSTR4 and only displayed mRNA transcripts of SSTR2, 3 and 5, however such possibility of cross-reaction cannot be ruled out from the discussion [66]. Consistent with previous studies, GRKs and β-arrestin exert critical role in stimulation and trafficking of GPCRs along with the modulation of downstream signaling including ERK phosphorylation [67]–[69]. Therefore differential recruitment of GRKs and β-arrestin in mono- vs cotransfectant cells is highly possible to exert such differential regulation of receptor function. Increased phospho-ERK5 expression was resulted due to nerve injury in dorsal root ganglion (DRG) neurons, whereas, knockdown of ERK5 resulted in a reduced neuropathic pain in DRGs and primary afferents [50]. We observed that SSTR4 activation maintained ERK5 phosphorylation in cotransfected cells albeit to a lesser degree in comparison to cells expressing SSTR4 alone. Thus, we predict that heteromeric complex of SSTR4/δOR by regulating ERK5 activation might play a significant role in nerve injury induced pain. Furthermore, our results also uncovered distinct role of Gi-proteins in ERK1/2 and ERK5 regulation as PTX treatment resulted in enhanced ERK1/2 activation and conversely blocked ERK5 activation in cotransfected cells.

In addition to ERKs, PI3K plays a critical role in the anti-nociception mediated by OR agonist that is blocked in presence of PI3K inhibitors [51], [70]. Previous studies have indicated that morphine induces anti-nociceptive responses that are associated with enhanced PI3K in periaqueductal grey matter (PAG) and upon supraspinal stimulation of µ-opioid receptors [70]. In addition, the activation of a PI3Kγ/AKT signaling modulates the ability of morphine to inhibit inflammatory nociception [71]. The peripheral anti-nociceptive effect of morphine was reversed upon pretreatment of rat hind paws with selective AKT inhibitor. Similarly, activation of AKT was observed in DRG neurons when treated with morphine, although this effect is largely dependent upon PI3K activation. In the present study, we showed that SSTR4 mediated activation of PI3K in monotransfected as well as cotransfected cells was sustained upon receptor specific agonist treatment either alone or in combination with δOR when compared to control. Results presented here also indicate that δOR stimulates AKT phosphorylation more significantly than SSTR4, whereas cotransfected cells display enhanced phospho-AKT specifically in presence of SSTR4 agonist. We argue that activation of PI3K/AKT seen in present study is direct physiologically relevant evidence for the role of SSTR4 in concert with δOR. Furthermore, mice lacking SSTR4 display sustained pain, loss in analgesic effect and susceptibility to inflammation [23].

In summary, the present study elucidates direct intra-membrane interaction between SSTR4 and δOR and uncovers a new mechanism for the possible drug targets in the management of pain. Our results provide new insight for the physiological response of SSTR4 and δOR in transfected cells and in native systems using combination of multiple techniques. Opiates are the most efficient drugs in alleviating pain but their sustained use in repeated manner are associated with addiction, dependence and withdrawal. Targeting SSTR4 and δOR heterodimers as a potential therapeutic approach can be used to minimize the risk of withdrawal while contributing to enhanced pain relief.

## Supporting Information

Figure S1 **Specificity of heteromeric complex formation in HEK-293 cells.** Cotransfected cells expressing SSTR4 and δOR were processed for Co-IP as indicated to determine the specificity of heterodimerization. Cells were treated with receptor specific agonist for 30 min at 37°C. The membrane fraction was isolated and solubilized with Tris-buffer and incubated with protein A/G agarose beads in absence of primary antibodies. The samples were electrophoresed, transferred to PVDF and incubated with δOR or SSTR4 (1∶250) specific primary antibodies (overnight at 4°C) and followed by incubation in secondary antibody (for 1 h at RT). Note that no expression of δOR or SSTR4 was detected in the immunoprecipitate prepared from cotransfected cells. The absence of bands at the expected molecular weights in either control or treated condition indicates the specificity of heterodimerization. Data are representative of three independent experiments.
(TIF)

Figure S2 **Microscopic Pb-FRET analysis in cells coexpressing δOR and SSTR4. (A)** Representative photomicrographs illustrating HA-SSTR4 (green) and cMyc-δOR (red) and colocalization (yellow) in cotransfected HEK-293 cells. Microscopic Pb-FRET was performed as described in Material and Methods. **(B and D)** A selection of photomicrographs illustrating photobleaching profile taken from the cells incubated with the donor alone **(B)** and in the presence of acceptor **(D)**. Histograms shown in panels **(C and E)** represent pixel by pixel analysis of time constant of donor in absence or presence of acceptor. The mean time constant shown in black calculated from a Gaussian distribution curve. Note the change in the time constant (τ) of donor in presence of acceptor, indicating interactions between SSTR4 and δOR at the cell surface in basal condition. Data are representative of three independent experiments whereas the number of cells analyzed per experiment ranged from 50–60.
(TIF)

Figure S3 **Changes in relative FRET efficiency upon Co-activation of SSTR4 and δOR.** HEK-293 cells expressing HA-SSTR4 (green) and cMyc-δOR (red) were treated with SST-14 (1 µM) and SB-205607 (10 nM) in combination for 15 min at 37°C. Combined activation of SSTR4 and δOR with receptor specific agonists displayed loss in relative FRET efficiency in comparison to control (Figure S2). Representative photomicrographs illustrating bleaching profile of the donor in the absence or presence of acceptor **(Panels B and D),** whereas, histograms shown in panels **C** and **E** represent pixel by pixel analysis of time constant of the donor alone or donor + acceptor respectively upon co-activation of the receptors. Data are representative of three independent experiments and 50–60 cells were analyzed per experiment.
(TIF)

Figure S4 **Effect of Gi inhibition on signaling pathways.** To ascertain the effect of Gi on the signaling pathways regulated by δOR and SSTR4, cotransfected cells were pretreated with PTX (100 ng/ml) for 16–18 h in DMEM at 37°C followed by treatment with receptor specific agonist as indicated for 15 min at 37°C. Cell lysates were processed for western blot analysis to analyze the expression levels of phospho-and total ERK1/2, ERK5, PI3K, AKT and PKA. Note the significant activation of PKA **(A)**, ERK1/2 **(B)** and AKT **(E)** along with complete loss of phospho ERK5 **(C)** and PI3K (D) upon pre-exposure of cells with PTX. β-Actin was used as the loading control. These results are the representative of three independent experiments.
(TIF)
