# Supplementary figures and images for: δ-Opioid Receptor and Somatostatin Receptor-4 Heterodimerization: Possible Implications in Modulation of Pain Associated Signaling
Source: PLoS One. 2014 Jan 8;9(1):e85193. doi: 10.1371/journal.pone.0085193 (PMC3885706; doi:10.1371/journal.pone.0085193)

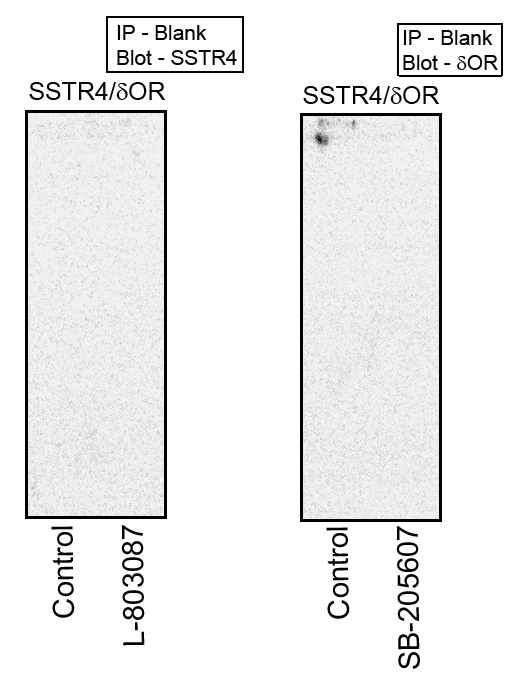

Supplement: Figure S1 — Specificity of heteromeric complex formation in HEK-293 cells. Cotransfected cells expressing SSTR4 and δOR were processed for Co-IP as indicated to determine the specificity of heterodimerization. Cells were treated with receptor specific agonist for 30 min at 37°C. The membrane fraction was isolated and solubilized with Tris-buffer and incubated with protein A/G agarose beads in absence of primary antibodies. The samples were electrophoresed, transferred to PVDF and incubated with δOR or SSTR4 (1∶250) specific primary antibodies (overnight at 4°C) and followed by incubation in secondary antibody (for 1 h at RT). Note that no expression of δOR or SSTR4 was detected in the immunoprecipitate prepared from cotransfected cells. The absence of bands at the expected molecular weights in either control or treated condition indicates the specificity of heterodimerization. Data are representative of three independent experiments. (TIF) [file pone.0085193.s001.tif]

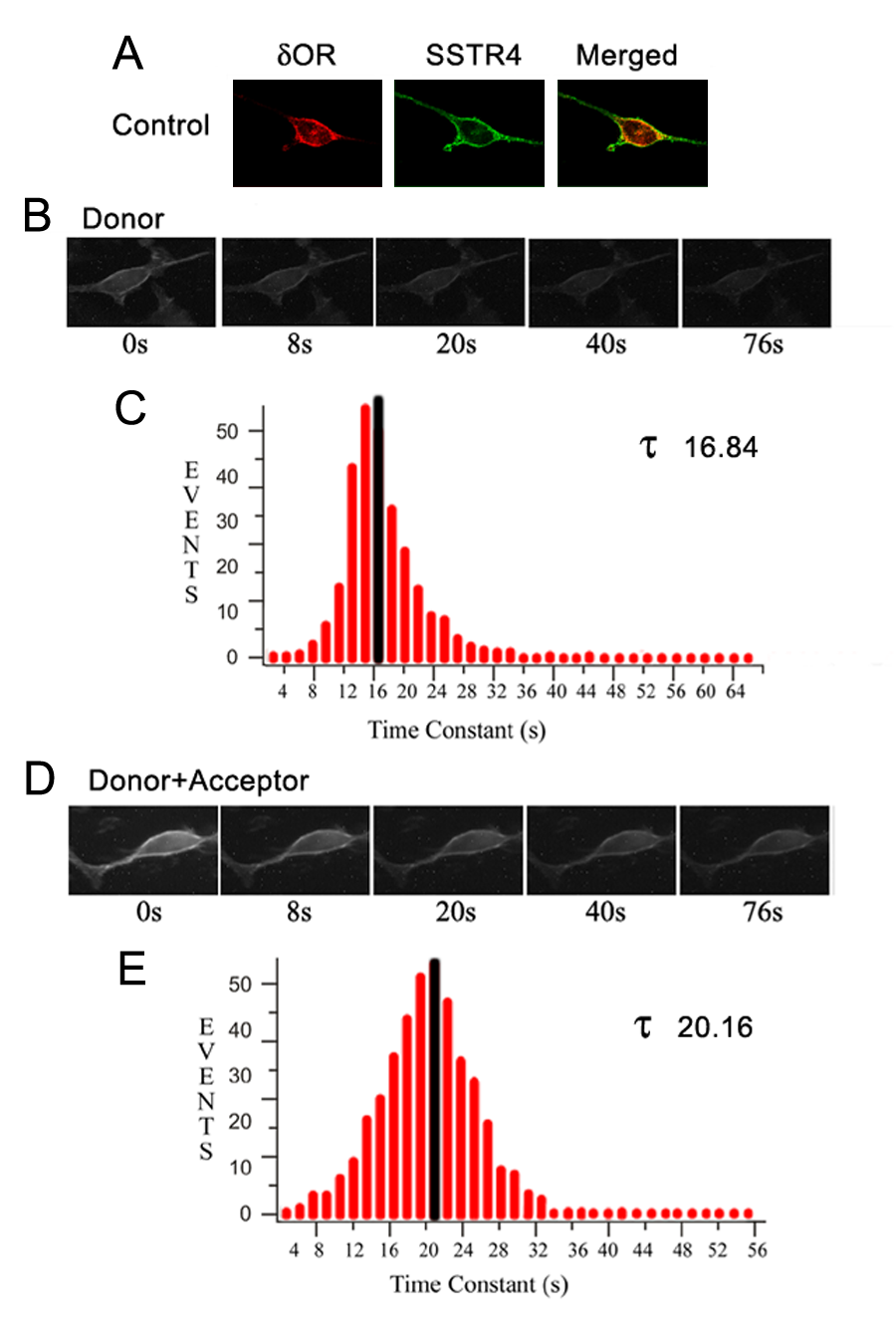

Supplement: Figure S2 — Microscopic Pb-FRET analysis in cells coexpressing δOR and SSTR4. (A) Representative photomicrographs illustrating HA-SSTR4 (green) and cMyc-δOR (red) and colocalization (yellow) in cotransfected HEK-293 cells. Microscopic Pb-FRET was performed as described in Material and Methods. (B and D) A selection of photomicrographs illustrating photobleaching profile taken from the cells incubated with the donor alone (B) and in the presence of acceptor (D). Histograms shown in panels (C and E) represent pixel by pixel analysis of time constant of donor in absence or presence of acceptor. The mean time constant shown in black calculated from a Gaussian distribution curve. Note the change in the time constant (τ) of donor in presence of acceptor, indicating interactions between SSTR4 and δOR at the cell surface in basal condition. Data are representative of three independent experiments whereas the number of cells analyzed per experiment ranged from 50–60. (TIF) [file pone.0085193.s002.tif]

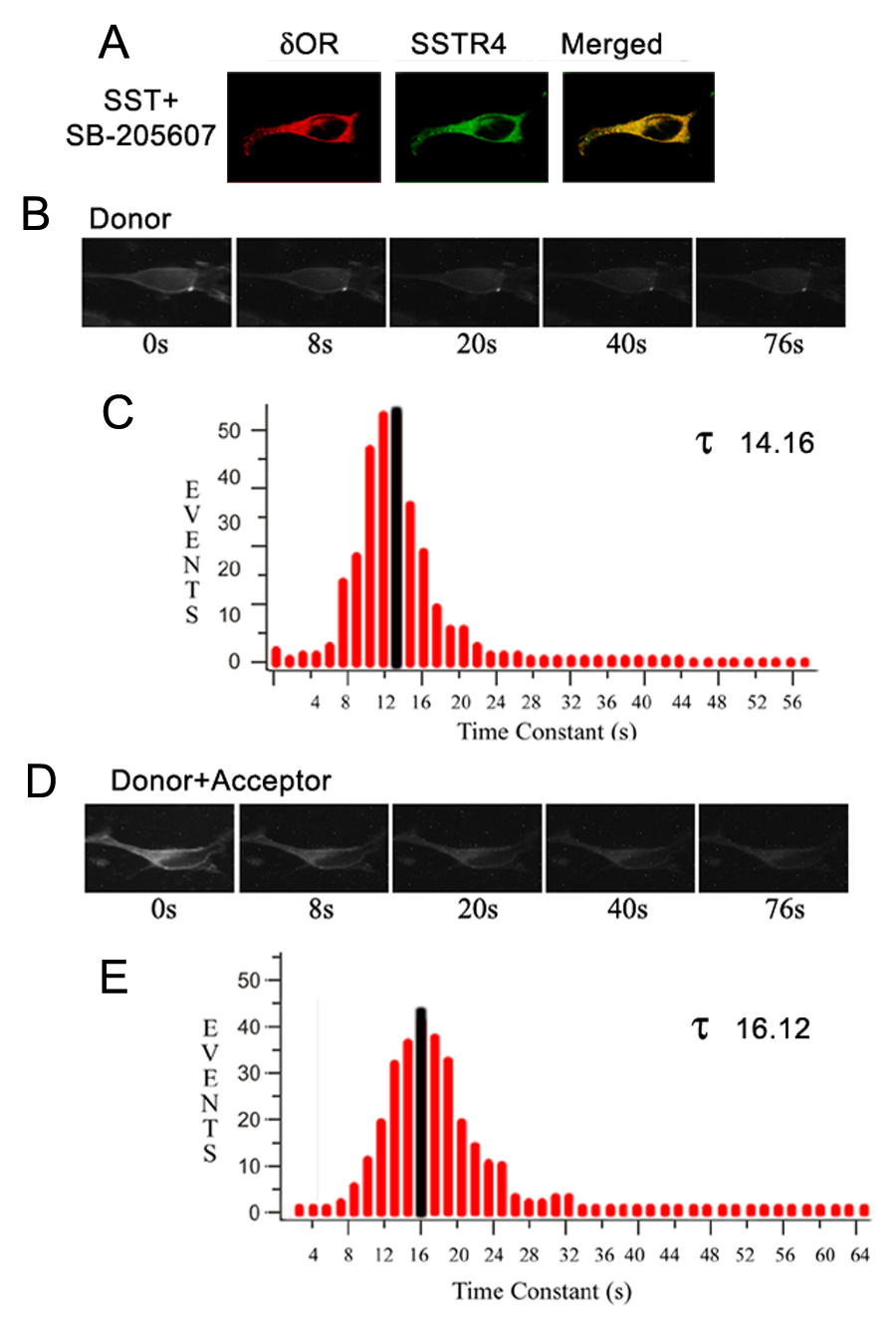

Supplement: Figure S3 — Changes in relative FRET efficiency upon Co-activation of SSTR4 and δOR. HEK-293 cells expressing HA-SSTR4 (green) and cMyc-δOR (red) were treated with SST-14 (1 µM) and SB-205607 (10 nM) in combination for 15 min at 37°C. Combined activation of SSTR4 and δOR with receptor specific agonists displayed loss in relative FRET efficiency in comparison to control (Figure S2). Representative photomicrographs illustrating bleaching profile of the donor in the absence or presence of acceptor (Panels B and D), whereas, histograms shown in panels C and E represent pixel by pixel analysis of time constant of the donor alone or donor + acceptor respectively upon co-activation of the receptors. Data are representative of three independent experiments and 50–60 cells were analyzed per experiment. (TIF) [file pone.0085193.s003.tif]

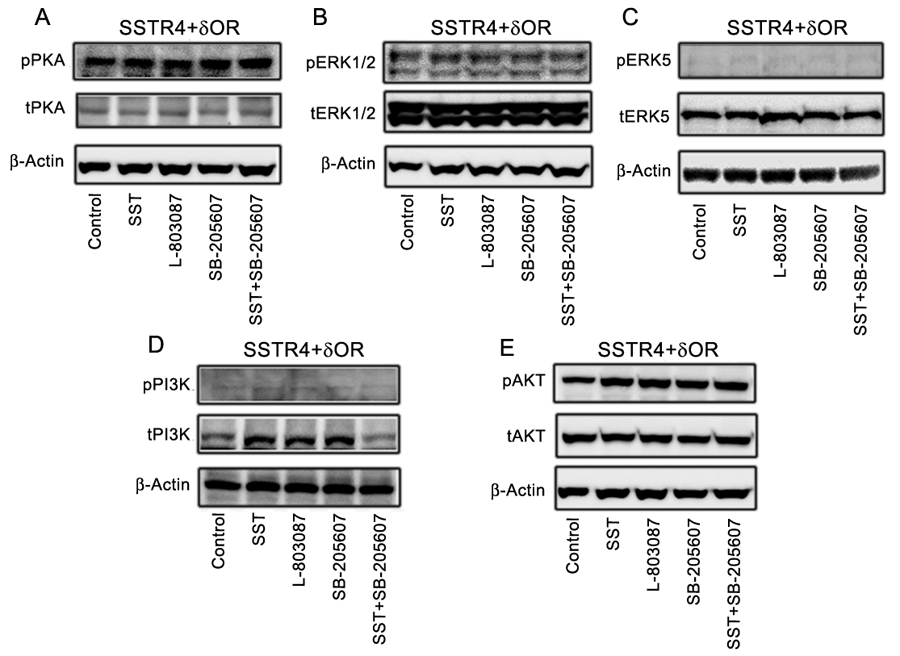

Supplement: Figure S4 — Effect of Gi inhibition on signaling pathways. To ascertain the effect of Gi on the signaling pathways regulated by δOR and SSTR4, cotransfected cells were pretreated with PTX (100 ng/ml) for 16–18 h in DMEM at 37°C followed by treatment with receptor specific agonist as indicated for 15 min at 37°C. Cell lysates were processed for western blot analysis to analyze the expression levels of phospho-and total ERK1/2, ERK5, PI3K, AKT and PKA. Note the significant activation of PKA (A), ERK1/2 (B) and AKT (E) along with complete loss of phospho ERK5 (C) and PI3K (D) upon pre-exposure of cells with PTX. β-Actin was used as the loading control. These results are the representative of three independent experiments. (TIF) [file pone.0085193.s004.tif]
